# Evaluation of potential aging biomarkers in healthy individuals: telomerase, AGEs, GDF11/15, sirtuin 1, NAD+, NLRP3, DNA/RNA damage, and klotho

**DOI:** 10.1007/s10522-023-10054-x

**Published:** 2023-07-31

**Authors:** Pavel Borsky, Drahomira Holmannova, Ctirad Andrys, Jan Kremlacek, Zdenek Fiala, Helena Parova, Vit Rehacek, Tereza Svadlakova, Svatopluk Byma, Otto Kucera, Lenka Borska

**Affiliations:** 1 Institute of Preventive Medicine, Faculty of Medicine in Hradec Kralove, Charles University, 50003 Hradec Kralove, Czech Republic; 2 Institute of Clinical Immunology and Allergology, University Hospital and Faculty of Medicine in Hradec Kralove, Charles University, 50003 Hradec Kralove, Czech Republic; 3 Institute of Medical Biophysics, Faculty of Medicine in Hradec Kralove, Charles University, 50003 Hradec Kralove, Czech Republic; 4 Institute of Clinical Biochemistry and Diagnostics, University Hospital and Faculty of Medicine in Hradec Kralove, Charles University, 50003 Hradec Kralove, Czech Republic; 5 Transfusion Center, University Hospital, 50003 Hradec Kralove, Czech Republic; 6 Institute of Physiology, Faculty of Medicine in Hradec Kralove, Charles University, 50003 Hradec Kralove, Czech Republic

**Keywords:** Aging, AGEs, GDF15, DNA/RNA damage, Sirtuin 1, NLRP3

## Abstract

Aging is a natural process of gradual decrease in physical and mental capacity. Biological age (accumulation of changes and damage) and chronological age (years lived) may differ. Biological age reflects the risk of various types of disease and death from any cause. We selected potential biomarkers of aging - telomerase, AGEs, GDF11 and 15 (growth differentiation factor 11/15), sirtuin 1, NAD^+^ (nicotinamide adenine dinucleotide), inflammasome NLRP3, DNA/RNA damage, and klotho to investigate changes in their levels depending on age and sex. We included 169 healthy volunteers and divided them into groups according to age (under 35; 35–50; over 50) and sex (male, female; male and female under 35; 35–50, over 50). Markers were analyzed using commercial ELISA kits. We found differences in values depending on age and gender. GDF15 increased with age (under 30 and 35–50 *p* < 0.002; 35–50 and over 50; *p* < 0.001; under 35 and over 50; *p* < 0.001) as well as GDF11 (35–50 and over 50; *p* < 0.03; under 35 and over 50; *p* < 0.02), AGEs (under 30 and 35–50; *p* < 0.005), NLRP3 (under 35 over 50; *p* < 0.03), sirtuin 1 (35–50 and over 50; *p* < 0.0001; under 35 and over 50; *p* < 0.004). AGEs and GDF11 differed between males and females. Correlations were identified between individual markers, markers and age, and markers and sex. Markers that reflect the progression of biological aging vary with age (GDF15, GDF11, AGEs, NLRP3, sirtuin) and sex (AGEs, GDF11). Their levels could be used in clinical practice, determining biological age, risk of age-related diseases and death of all-causes, and initiating or contraindicating a therapy in the elderly based on the patient’s health status.

**Supplementary Information:**

The online version contains supplementary material available at 10.1007/s10522-023-10054-x.

## Introduction

Aging can be defined as a gradual, irreversible loss of physiological integrity leading to deterioration of tissue/organ function, vulnerability, and death. Aging is associated with a range of molecular and cellular changes that are accompanied by the changes in the production of specific products, biomarkers of aging (Preston and Biddell^2021^).

The most important features of aging are genomic instability often caused by oxidative stress, loss of proteostasis, AGE (advanced glycation end products) production, mitochondrial dysfunction, telomere shortening, altered intracellular communication, deregulation of nutrient sensing, cell senescence, depletion of stem cell function, DNA methylation/epigenetic modification, etc. (Jylhävä et al. 2017; Razgonova et al. 2020).

There may be a difference between chronological and biological age. Biological age can be evaluated by analysis of a variety of markers. We carefully selected markers that reflect the processes associated with aging. These are telomerase, AGE, GDF 11 and 15 (growth differentiation factor 11/15), sirtuin 1, NAD^+^ (nicotinamide adenine dinucleotide), inflammasome NLRP3 (NOD-, LRR- and pyrin domain-containing protein 3), DNA/RNA damage, and klotho (Hartmann et al. 2021; López-Otín et al. 2022).

Telomerase is a ribonucleoprotein complex composed of the enzyme reverse transcriptase protein TERT and the crucial RNA template to protect the ends of chromosomes (telomeres) from shortening during DNA replication. Gradual telomere shortening and a decrease in telomerase activity are associated with aging (Vaiserman and Krasnienkov^2021^). It is thought that restoration of telomerase function can reverse cell aging. On the other hand, increased telomerase activity and telomere elongation can lead to genomic instability, as seen in some types of tumors (McNally et al. 2019; Liu et al. 2016).

AGEs are advanced glycation end-products (proteins, lipids) that are formed by a non-enzymatic glycation reaction with damaging potential. They can promote oxidative stress, inflammation, or cell death (Rungratanawanich et al. 2021) Their concentration in the organism slowly increases during physiological aging. Pathologies such as inflammation, metabolic diseases, excessive alcohol consumption, smoking, and a high-caloric diet can accelerate the production of AGEs and aging (Chaudhuri et al. 2018).

GDF11 and 15 are members of a TGFβ family. GDF11 is known to be a rejuvenating factor that is necessary during development and decreases with age. It has anti-aging, proangiogenic and proneurogenic properties and thus supports tissue regeneration (Zhang et al. 2021; Song et al. 2022). GDF15 depends on stress stimuli (presence of metabolic syndrome, cardiovascular diseases, diabetes and aging) and reflects mitochondrial dysfunction. It increases with age and is associated with decreased muscle performance and increased inflammation (Conte et al. 2020; Wischhusen et al. 2020; Fujita et al. 2016)

Sirtuin 1 (SIRT1) is NAD-dependent deacetylase sirtuin-1. Its activity depends on the level of NAD and the presence of inflammation. Chronic low-grade inflammation is common for aging. Inflammation is associated with NF-κB activation, and this transcriptional factor inhibits SIRT1. The activity of SIRT1 is also limited by the decrease in the availability of NAD^+^ with age (Kane and Sinclair 2018; Imai and Guarente 2014). SIRT1 has a protective role in the organism, protecting against metabolic syndrome, obesity, cardiomyopathy, and the decline in vascular endothelial function. Sirtuin 1 and NAD are also required for telomere maintenance (Palacios et al. 2010). NAD + is a coenzyme in redox reactions, including reactions in the tricarboxylic acid cycle (ichiro Imai and Guarente 2014).

Chronic inflammation is associated with inflammaging in which inflammasome NLRP3 plays a crucial role. NLRP3 is an intracellular structure responsible for the processing of inactive IL-1 and IL-18 into active proinflammatory cytokines. The assembly of NLRP3 is induced in the presence of substrates that form during aging (AGE, amyloid β, α-synuclein, oxidized LDL) or during infection (Gritsenko et al. 2020). It was shown that inhibition of NLRP3 activation prolonged the lifespan of mice in a murine model of progeria (González-Dominguez et al. 2021).

Aging and inflammation are accompanied by an increase in the production of reactive oxygen species (ROS), which interact with vital macromolecules such as proteins, lipids, and even DNA and RNA. These interactions can lead to irreversible DNA/RNA damage and genomic instability and can also accelerate aging (Gonzalez-Hunt et al.  2018).

Klotho is a crucial factor that regulates numerous pathways involved in aging (regulation of phosphate metabolism, insulin and Wnt signaling, p53/p21, cAMP, mTOR, protein kinase C, TGFβ. Its expression declines during aging. A decrease in klotho levels is associated with endothelial dysfunction, intima hyperplasia, arterial stiffness, hypertension, diabetes, etc. (Buchanan et al. 2020; Clemens et al. 2021).

The aim of our study was to investigate how the values of selected markers are influenced by age and gender. Knowledge of the values of markers of aging and the dynamics of their changes not only in relation to aging but also in relation to sex may help to understand the changes that accompany aging and intersex differences during aging. This could also be used in clinical practice, in establishing different approaches to women and men in the diagnosis and treatment especially aging-related diseases.

## Materials and methods

Total 169 individuals were enrolled in our study. Persons with any inflammatory diseases, pregnancy, and those using nonsteroidal, anti-inflammatory medications or diagnosed chronic diseases were excluded. The recruitment process of the study took place at the Transfusion clinic of the University Hospital in Hradec Kralove. Participants were eligible to be included in the study only if they met the criteria of becoming volunteer plasma or blood donors. Exclusion criteria included: diabetes mellitus, hepatitis B or C, extrapulmonary TBC, severe blood disease, malignant tumors, myocardial infarction, stroke, psychiatric disease, transplantation. TBC, rheumatic fever in past two years. Mononucleosis, hepatitis A, sepsis, polytrauma, risky sexual contact during last year. Borreliosis, toxoplasmosis, surgery, anesthesia, childbirth, interruption, severe accidents, arthroscopy, gastro/colono/cystoscopy, catheterization, thrombosis, medication affecting hemocoagulation, gastric ulcers, tattoos, piercing, acupuncture, sexually transmittable disease, blood transfusion in past six months. Viral infection with fever, antibiotic treatment, vaccination for hepatitis B, flu, typhus, cholera, yellow fever, minor surgery without anesthesia, breastfeeding, lyme in past four weeks. Vaccination for covid-19, viral infection without fever, coughing or sneezing, tooth extraction, cold sore on the mouth in the past two weeks.

All subjects signed the informed consent before participating in the study. The study was conducted in accordance with the Declaration of Helsinki, and the protocol was approved by the Ethics Committee of the Faculty Hospital in Hradec Kralove, Czech Republic (Project identification code PROGRES Q40-09 and Q40-10, reference number 201,705 I83P, date 2 May 2017).

Participants were divided into groups and subgroups:

- according to age: under 35 (*n* = 57), 35–50 (*n* = 59), and over 50 (*n* = 53).
- according to sex: male (*n* = 85, female (*n* = 84) and.
- according to sex and age: males under 35 (*n* = 27), 35–50 (*n* = 31), over 50 (*n* = 31), females under 35 (*n* = 30), 35–50 (*n* = 28), over 50 (*n* = 26). 

### Biochemical parameters

Biochemical parameters estrogen, progesterone, DHEA, and testosterone were measured in serum from blood samples withdrawn from the cubital vein using standard laboratory methods at the Institute of Clinical Biochemistry and Diagnostics (FN and LF UK in Hradec Kralove).

### Blood sample collection

Peripheral blood samples were collected from the cubital vein of all participants at the Transfusion center, University Hospital, Hradec Kralove, from the cubital vein by using BD Vacutainer sampling tubes. Blood serum was isolated by centrifugation and the samples were stored at − 70 °C until analysis. Repeated thawing and freezing were avoided.

### Biomarker analyses

All biomarkers were evaluated using commercial ELISA kits according to the manufacturer’s instructions and the absorbance values were read at 450 nm on a Multiskan RC ELISA reader (Thermo Fisher Scientific, Waltham, MA, USA).

- Telomerase: Human Telomerase (TE) ELISA Kit ELISA Kit (Cusabio, Houston, Texas, USA); samples were 2-fold diluted. The detection range of the kit was from 0.31 to 40 ng/ml.
- Klotho: using Human Klotho ELISA Kit (Cusabio, Cloud-Clone Corp, Katy, Texas, USA); samples were not diluted. The detection range of the kit was from 0.156 to 10 ng/ml.
- NLRP3: Human NALP/NLRP3 ELISA Kit (LifeSpan BioSciences, Inc. Seattle, USA); samples were not diluted. The sensitivity of the kit was from 0.313 to 40 ng/ml.
- AGEs: Human Advanced Glycation End Products (Agens) ELISA Kit (Cusabio, Houston, Texas, USA); samples were not diluted. The detection range of the kit was from 0.78 to 50 µg/ml.
- GDF11: Human GDF11/GDF11 ELISA Kit (LifeSpan BioSciences, Inc. Seattle, USA); samples were not diluted. The detection range of the kit was from 7.8 to 1000 pg/ml.
- GDF15: Quantikine ELISA Human GDF15 Kit (R&D Systems, MN, USA); samples were 4-fold diluted. The detection range of the kit was from 93,6 to 6000 pg/ml.
- Sirtuin-1: Human SIRT 1/Sirtuin 1 ELISA Kit (LifeSpan BioSciences, Inc. Seattle, USA); samples were 50-fold diluted. The detection range of the kit was from 3.9 to 250 ng/ml. Results were converted from pg/ml to ng/ml due to smaller numbers.
- DNA/RNA damage: DNA/RNA Oxidative Damage EIA Kit (Cayman Chemical Company, USA); samples were 100-fold diluted. The detection range of the kit was from 10 to 30 000 pg/ml 8-hydroxy 2-deoxy guanosine.
- NAD: Enzyme-linked Immunosorbent Assay Kit For Nicotinamide Dinucleotide (NAD) (Cloud-Clone Corp. (Katy, Texas, USA); samples were 20-fold diluted. The detection range of the kit was from 2400 to 200 000 ng/ml.

### Statistical analysis

The data were statistically processed by the R software version 3.6.1 “nortest”, “compute.es”, and “ggplot2”. Based on normality distribution evaluation (the Anderson–Darling test), parametric or nonparametric tests were used. Relationship between parameters were evaluated either by Pearson’s or by Spearman’s correlation test. Differences among groups were assessed using Student’s t or Wilcoxon rank-sum test. The null hypothesis was rejected when the probability level (p) reached below 0.05 (the alpha level).

## Results

### Demographic data, hormonal parameters

The 169 participants were enrolled in our study: 85 male and 84 female. They were divided into groups according to their age and subgroups according to sex and age.

The groups according to the age:

- under 35 (*n* = 57; 30 females and 27 males; median age 28.14 years, min. 19.63, max. 35 years),
- 35–50 (*n* = 59; 28 female and 31 males; median 42.43, min. 35.4, max. 49.5 years),
- over 50 (*n* = 53; 26 females and 27 males; median 55.27, min. 50.2, max. 65.92 years) years of age.

The groups according to the sex: 84 females (median 42, min. 19, max. 65 years) and 85 males (median 43.5, min.20.36, max. 66 years).

The subgroups according to the age and sex:

- males and females under 35 (*N* = 27; median 29.3 years and *N* = 30; 27.5 years).
- males and females 35–50 (*N* = 31; median 43.5 years; *N* = 28; 41.4 years).
- males and females over 50 (*N* = 27, median 55.9 years; *N* = 26; median 55.3 years).

There were 20 smokers and 128 nonsmokers. The levels of any analyzed markers did not differ between smokers and non-smokers in female and male and among age subgroups (Table 1).

**Table 1:** Difference in the levels of aging biomarkers by age

	N	Median	Q1	Q3	Min.	Max.	*p* value under 35/35–50	*p* value 35–50/over50	*p* value under 35/over50
**Telomerase** ng/ml									
Under 35	57	4.515	2.015	12.167	0.001	30.516	NS	NS	NS
35–50	59	8.220	2.460	17.440	0.130	32.677			
Over 50	53	4.056	1.423	17.700	0.093	31.518			
**AGE** µg/ml (advanced glycation end products)									
Under 35	57	10.909	7.592	14.401	2.822	38.425	*p* < 0.01	NS	NS
35–50	59	15.293	10.469	20.682	3.176	49.500			
Over 50	53	12.125	8.103	21.903	2.097	37.210			
**DNA/RNA** pg/ml									
Under 30	57	7228	4311	12,504	127	42,967	NS	NS	NS
35–50	59	9139	4314	22,517	0	48,521			
Over 50	53	6590	3020	12,249	0	44,900			
**GDF15** pg/ml (growth differentiation factor 15)									
Under 30	57	240.713	193.485	272.795	88.390	599.508	*p* < 0.01		*p* < 0.001
35–50	59	286.333	326.432	343.419	117.711	460.993		*p* < 0.001	
Over 50	53	384.400	301.195	473.814	202.026	819.477			
**GDF11** pg/ml (growth differentiation factor 11)									
Under 30	57	5.580	3.816	8.394	1.296	40.082	NS		*p* < 0.05
35–50	59	4.818	3.102	9.204	0.958	83.721		*p* < 0.05	
Over 50	53	8.618	4.835	11.379	1.811	88.427			
**NAD** ng/ml (nicotinamide adenine dinucleotide)									
Under 30	57	731.8	410.0	934.20	29.5	1694.8	NS	NS	NS
35–50	59	620.1	303.5	914.45	29.3	2017.8			
Over 50	53	803.9	803.9	1056.5	25.4	1842.1			
**Sirtuin** ng/ml									
Under 30	57	252.00	191.00	378.00	79	635	NS		*p* < 0.01
35–50	59	294.50	232.00	362.25	64	1075		*p* < 0.0001	
Over 50	53	192.50	117.75	244.00	1	870			
**Klotho** ng/ml									
Under 30	57	0.161	0.099	0.277	0.011	1.055	NS	NS	NS
35–50	59	0.175	0.108	0.337	0.012	4.382			
Over 50	53	0.253	0.129	0.416	0.008	2.536			
**NLRP3** ng/ml (NOD-, LRR- and pyrin domain-containing protein 3)									
Under 30	57	0.008	0.005	0.012	0.001	0.116	NS		*p* < 0.05
35–50	59	0.010	0.005	0.020	0.001	0.120		NS	
Over 50	53	0.012	0.007	0.035	0.001	0.093			

BMI did not differ between the subgroups divided according to age but was higher in the male group and in the male under 35 compared the female group and female under 35 (*p* < 0.003; *p* < 0. 03).

As expected, the estrogen levels were significantly higher in females and decreased significantly with age in all participants, except the levels of estrogen were higher in males compared to females in over 50 group (*p* < 0.002).

The levels of progesterone were higher in females and significantly decreased with age in all participants. Surprisingly, there was not difference in progesterone levels in the male and female groups over 50.

The testosterone levels were significantly higher in the male groups and did not significantly differ between 35 and 50 years of age and over 50 but were lower in the subject under 35 compared to older males. The levels were higher in men at any age compared to the similarly old women.

The levels of DHA decreased with age in all participants. The levels of DHEA were higher only in males over 50 (*p* < 0.0001) compared to women, while there was no difference between males and females at age under 35 and 35–50. A table depicting BMI and hormonal levels is available in supplementary.

### Parameters of aging

#### Telomerase

The levels of telomerase did not differ among age subgroups. There was no difference between the subgroups due to sex and age (Tables 1 and 2).

**Table 2:** Descriptive data and p-values testing difference between male and female of aging biomarkers

	N	Median	Q1	Q3	Min	Max	*p* value
**Telomerase** ng/ml							
Female	84	4.86	1.19	14.49	0.02	30.52	NS
Male	85	7.98	2.31	15.85	0.00	32.68	
Female < 35	30	3.66	1.26	8.68	0.02	30.52	NS
Male < 35	27	6.48	2.27	12.27	0.00	29.04	
Female 35–50	28	5.89	1.13	16.05	0.13	28.90	NS
Male 35–50	31	8.44	4.60	19.89	0.22	32.68	
Female > 50	26	4.70	1.36	13.77	0.09	26.72	NS
Male > 50	27	3.56	1.92	15.27	0.90	31.52	
**AGE** µg/ml (advanced glycation end products)							
Female	84	10.63	7.46	16.14	2.10	38.3	*p* < 0.001
Male	85	14.61	9.69	21.37	2.98	49.5	
Female < 35	30	10.25	6.25	12.65	2.82	38.30	NS
Male < 35	27	12.11	8.65	15.89	2.98	38.42	
Female 35–50	28	11.00	8.22	17.78	3.18	25.97	*p* < 0.0001
Male 35–50	31	19.35	14.11	23.15	8.10	49.50	
Female > 50	26	9.49	7.55	23.01	2.10	37.21	NS
Male > 50	27	12.98	8.61	20.47	5.83	31.41	
**DNA/RNA damage** pg/ml							
Female	84	6591	3727	11,327	0.00	41,276	NS
Male	85	9134	4337	20,331	0.16	48,521	
Female < 35	30	6777	3893	10,840	201.29	35,820	NS
Male < 35	27	8067	5068	14,286	127.16	42,967	
Female 35–50	28	6920	3698	21,127	285.51	41,110	NS
Male 35–50	31	11,381	7226	26,066	0.16	48,521	
Female > 50	26	6591	3236	10,650	0.00	41,276	NS
Male > 50	27	6759	2593	15,780	263.47	44,900	
**GDF15** pg/ml (growth differentiation factor 15)							
Female	84	268.88	224.70	352.92	88.39	800.54	NS
Male	85	298.85	233.24	370.48	117.71	819.48	
Female < 35	30	242.16	185.42	266.03	88.39	599.51	NS
Male < 35	27	230.76	195.70	278.68	144.52	362.43	
Female 35–50	28	262.26	228.44	337.93	148.12	460.99	NS
Male 35–50	31	304.33	243.32	343.42	117.71	428.43	
Female > 50	26	344.70	299.38	465.06	202.03	800.54	NS
Male > 50	27	410.46	328.74	472.01	222.08	819.48	
**GDF11** pg/ml (growth differentiation factor 11)							
Female	82	6.95	3.84	10.40	0.96	88.43	NS
Male	83	4.97	3.44	9.61	1.30	83.72	
Female < 35	30	5.58	3.74	8.32	1.78	40.08	NS
Male < 35	27	5.58	3.90	8.64	1.30	39.32	
Female 35–50	27	7.27	3.43	10.76	0.96	50.42	*p* < 0.05
Male 35–50	31	3.68	2.96	6.16	1.64	83.72	
Female > 50	25	8.73	6.18	11.06	2.64	88.43	NS
Male > 50	25	8.28	4.72	11.81	1.81	18.72	
**NAD** ng/ml (nicotinamide adenine dinucleotide)							
Female	84	787.6	416.78	1066.65	29.3	2017.8	NS
Male	85	639.2	341.8	891.1	25.4	1584.5	
Female < 35	30	845.3	529.47	1075.2	29.5	1694.8	NS
Male < 35	27	602.6	404.9	845.5	38.5	1231.2	
Female 35–50	28	646.0	394.48	953.85	29.3	2017.8	NS
Male 35–50	31	620.1	264.8	858.2	37.8	1249.0	
Female > 50	26	793.9	433.68	1054.48	51.2	1841.1	NS
Male > 50	27	803.9	323.75	1028.75	25.4	1584.5	
**Sirtuin 1** ng/ml							
Female	82	243	179	376.5	41	1075	NS
Male	83	258	186	331.5	1	870	
Female < 35	30	277.5	193.5	395.75	115	605	NS
Male < 35	27	241.0	187.5	331.50	79	635	
Female 35–50	27	318	210.5	422	64	1075	NS
Male 35–50	31	293	257.5	343	148	530	
Female > 50	25	193	114	229	41	478	NS
Male > 50	25	192	153	317	1	870	
**Klotho** ng/ml							
Female	84	0.21	0.14	0.37	0.01	4.38	NS
Male	85	0.17	0.10	0.35	0.01	3.50	
Female < 35	30	0.17	0.13	0.28	0.01	01.05	NS
Male < 35	27	0.14	0.09	0.26	0.02	0.57	
Female 35–50	28	0.18	0.11	0.35	0.01	4.38	NS
Male 35–50	31	0.17	0.11	0.32	0.01	3.50	
Female > 50	26	0.26	0.19	0.41	0.05	2.36	NS
Male > 50	27	0.24	0.12	0.41	0.01	2.54	
**NLRP3** ng/ml (NOD-, LRR- and pyrin domain-containing protein 3)							
Female	84	0.01	0.01	0.02	0	0.12	NS
Male	85	0.01	0.00	0.02	0	0.12	
Female < 35	30	0.01	0	0.01	0	0.07	NS
Male < 35	27	0.01	0	0.01	0	0.12	
Female 35–50	28	0.01	0.01	0.02	0	0.12	NS
Male 35–50	31	0.01	0.00	0.02	0	0.12	
Female > 50	26	0.01	0.01	0.02	0	0.08	NS
Male > 50	27	0.01	0.01	0.05	0	0.09	

### AGE

**Fig. 1:**
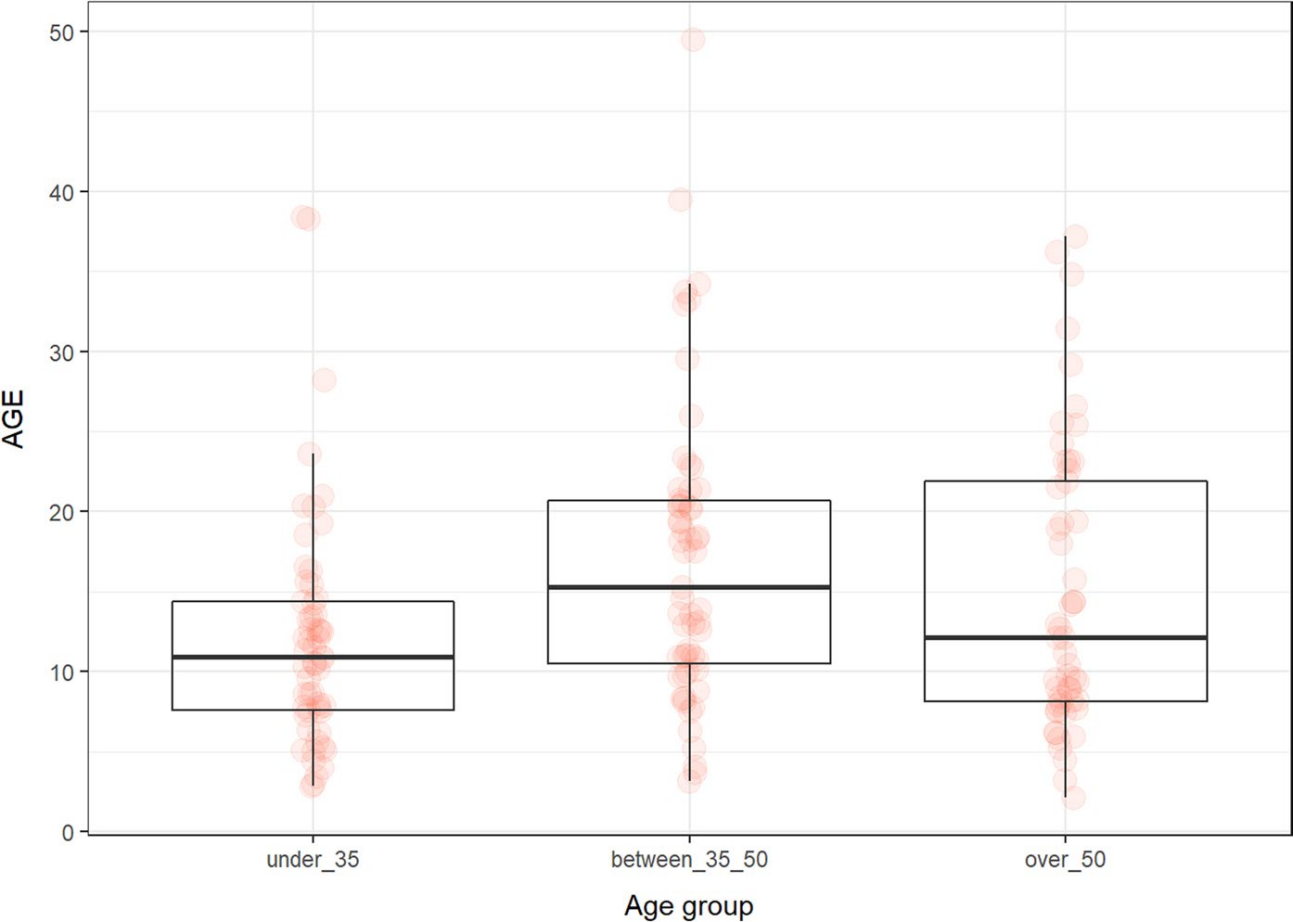
The levels of AGEs (µg/ml) in the groups of participants according to age

**Fig. 2:**
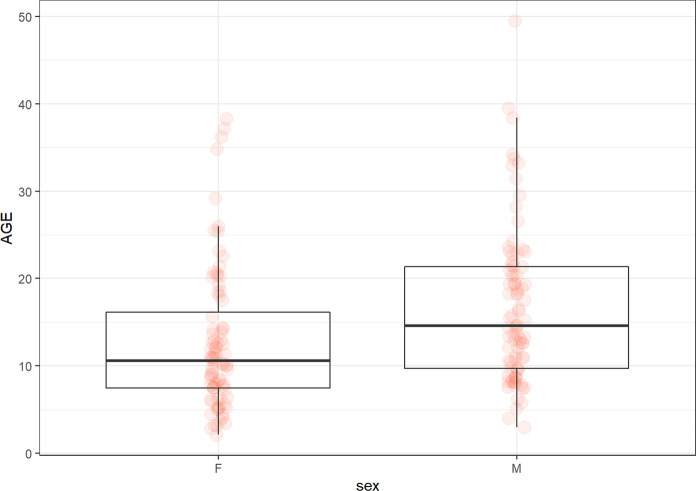
The levels of AGEs (µg/ml) in the male and female groups

The levels of AGE depended on chronological age. The highest were in individuals in 35–50 group. The significant difference was detected between under 30 and 35–50 group (*p* < 0.005). Interestingly, there were differences in the sexes. The levels of AGE were significantly higher in males compared to females (*p* < 0.001). The main difference was in the subgroup of 35–50 (*p* < 0.0004) (Tables 2 and 3; Figs. 1 and 2).

### DNA/RNA damage

The levels of DNA/RNA damage did not differ between groups and any subgroups.

### GDF15

**Fig. 3:**
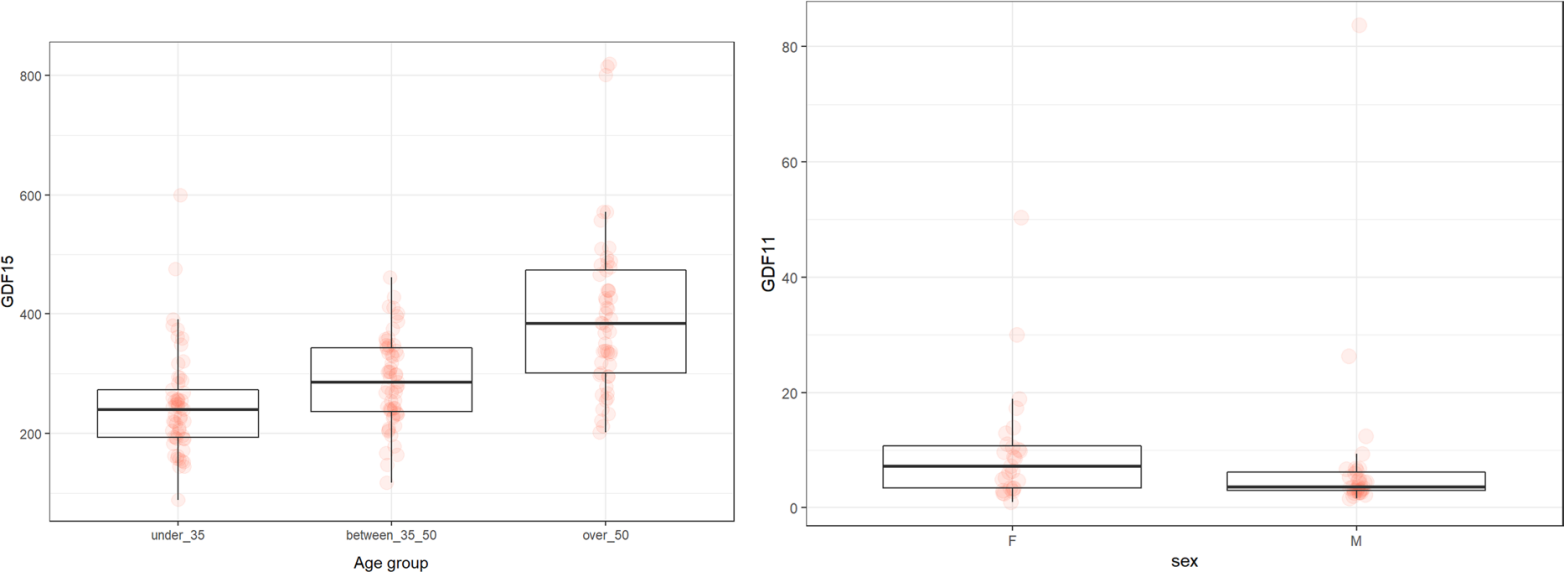
The levels of GDF15 (pg/ml) in the groups of participants according to age and sex

The levels of GDF15 significantly differ among the groups There were significant differences between under 35 and 35–50 (*p* < 0.002), 35–50 and over 50 (*p* < 0.001) and under 35 and over 50 (*p* < 0.001). The levels of GDF15 did not depend on sex in any age subgroups (Fig. 3).

### GDF11

**Fig. 4:**
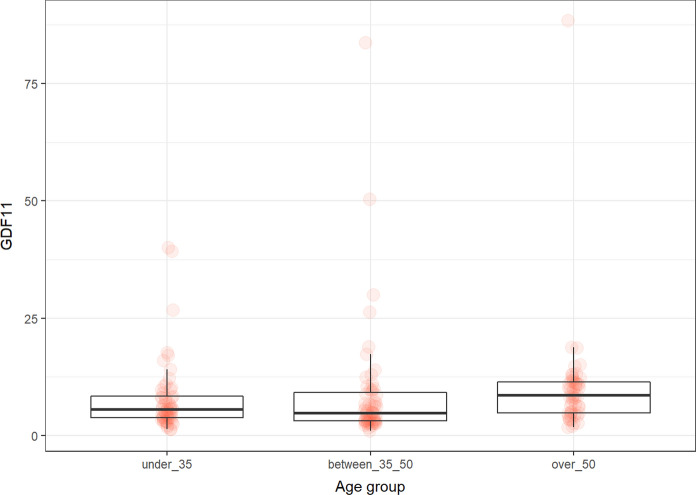
The levels of GDF11 (pg/ml) in the groups of participants according to age

The levels of GDF11 significantly differed between 35 and 50 and over 50 (*p* < 0.03) and under 35 and over 50 (*p* < 0.02). We also found the difference between the male and female in the 35–50 subgroup (*p* = 0.03). The lower levels were in males (Fig. 4).

### NAD and Klotho

The levels were similar in all groups and subgroups, thus did not depend on age and sex.

### Sirtuin 1

**Fig. 5:**
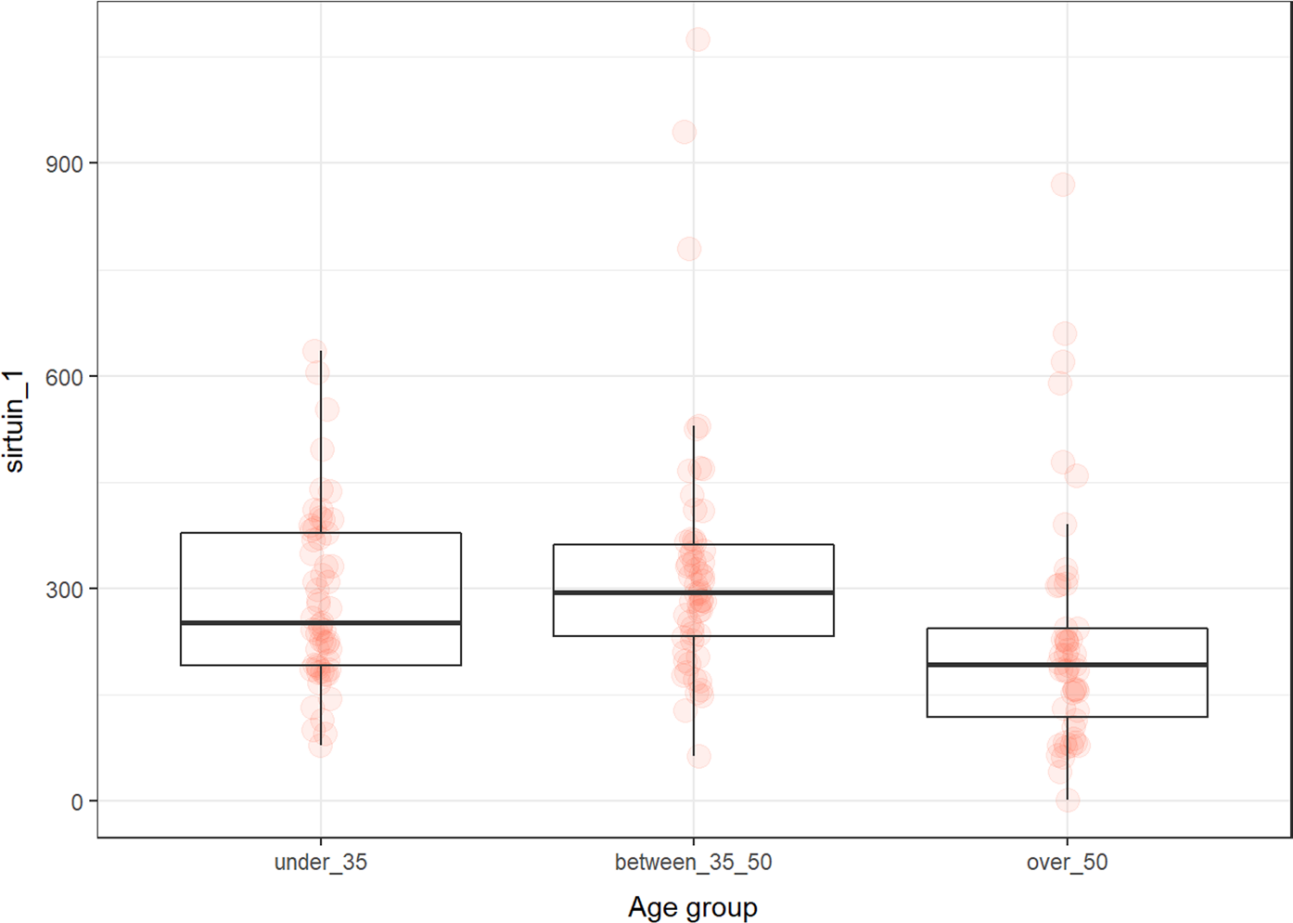
The levels of sirtuin 1 (ng/ml) in the groups of participants according to age

The levels of sirtuin 1 changed dependently on age. There were significant differences between 35 and 50 and over 50 (*p* < 0.0001) and under 35 and over 50 (*p* < 0.004) (Fig. 5). The levels were not influenced by sex.

### NLRP3

**Fig. 6:**
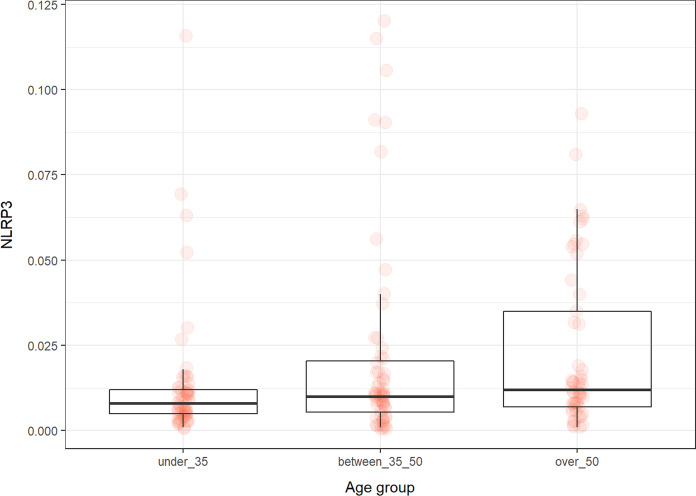
The levels of NLRP3 (ng/ml) in the groups of participants according to age

The levels of NLRP3 differ only between the group of under 35 and over 50 (*p* < 0.03) and did not depend on sex (Fig. 6).

### Correlations

**Fig. 7:**
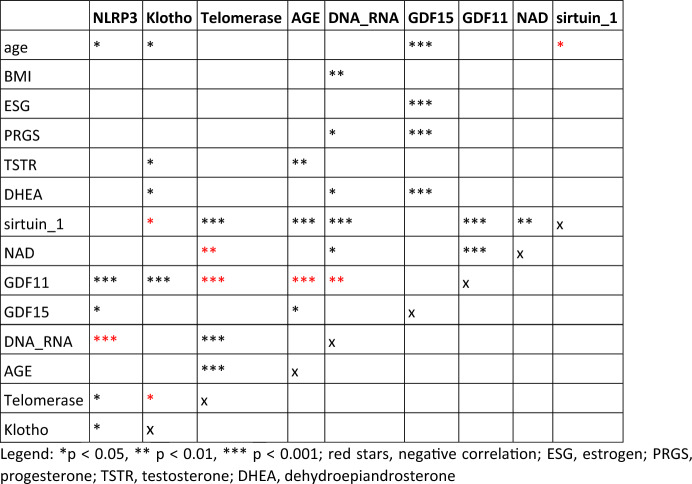
Correlation among markers in the male and female group

We performed analysis of relationships of all measured parameters for all participants and for males and females, non-dependently on age. In our cohort, we found significant correlations among a variety of measured parameters in the group of all participants. Markers representing aging correlated with each other, in addition to sex hormones and age (Table 3; Fig. 7). There were significant differences in the correlations between the male and female groups. Some correlations were documented only in one group. In women, we more often observed the dependence of aging markers on sex hormones. In men, NLRP3 was significantly more correlated with more parameters than in women, and correlations between klotho and some parameters were presented only in men (Table 4; Fig.  8 in supplementary).

**Table 3:** Correlations between parameters

	Spearman rho	*p* value
NLRP3		
Age	0.186	0.0157
GDF11	0.284	0.219e-03
GDF15	0.167	0.030
DNA/RNA	−0.320	1.659e-05
KLOTHO		
Age	0.183	0.017
Sirtuin 1	−0.168	0.031
GDF11	0.294	0.129e-03
Telomerase	−0.154	0.046
Testosterone	−0.159	0.039
DHEA	−0.176	0.022
TELOMERASE		
Sirtuin	0.656	1.177e-21
NAD	−0.202	0.008
GDF11	−0.351	3.704e-06
AGE		
Sirtuin	0.352	3.367e-06
GDF11	−0.294	0.124e-03
GDF15	0.163	0.035
Testosterone	0.239	0.002
DNA/RNA		
NAD	0.154	0.046
Sirtuin	0.313	4.296e-05
GDF11	−0.201	0.010
Progesterone	−0.162	0.035
DHEA	0.188	0.014
GDF15		
Age	0.570	6.477e-16
Estrogen	−0.285	0.171e-03
Progesterone	−0.307	4.807e-05
DHEA	−0.3253	1.632e-05
GDF11		
NAD	0.317	3.352e-05
Sirtuin		
Age	−0.188	0.016

## Discussion

Aging is a physiological process that can be accelerated by numerous stimuli. Aging processes include telomere shortening, disruption of protein synthesis and degradation, mitochondrial damage, disruption of intercellular communication, stem cell damage, senescence, inflammaging, epigenetic changes, altered nutrient sensing etc. (Hartmann et al. 2021; Colloca et al. 2020). If these processes are accelerated, the biological and chronological age may differ substantially and morbidity and mortality may increase. Biological age can be monitored using specific markers that reflect the processes mentioned above.

We measured nine selected markers in volunteers divided into groups according to age, sex, and sex and age to find whether these markers are affected by age and sex.

Our first evaluated marker was telomerase, the enzyme involved in the regulation of telomere length. Interestingly, we did not detect any difference in the levels of telomerase between any groups. Our results are in agreement with those of Hertzog et al. , who showed that the levels of telomerase did not differ between young (30–40 years) and older (more than 90 years) healthy volunteers. They detected a slight decrease in telomerase levels in younger people depending on age while there was an increase in the levels of telomerase in the older group depending on age (Hertzog et al. 2021). Higher or stable telomerase levels in the elderly may predict longevity. However, the increase of telomerase levels in younger subjects is associated with numerous pathological conditions such as cancer or severe chronic kidney disease (Noureen et al. 2021; Kidir et al. 2017). Short-term increases in telomerase levels can also occur under physiological conditions during moderate and high physical activity (Niedrist et al. 2022). Looking at the parameters that correlated with telomerase, there is a significant negative correlation with GDF11 in the group of all participants, the male and female group. However, various studies have shown that there is a positive relationship between GDF11 and telomerase activity. In the study by Chen et al. , mice with ischemia-reperfusion injury overexpressing GDF11 in the myocardium had higher telomerase activity (Chen et al. 2021). Wang et al. showed that the loss of GDF11 significantly downregulated telomerase activity (Wang et al. 2021). Zhao et al. documented the positive effect of telomerase on GDF11 rejuvenation function in patients after myocardial infarction (Zhao et al. 2019). The difference in our results may be due to the fact that our study involved healthy subjects, whereas in the cited studies the authors worked with subjects with medical pathologies.

A positive correlation was found between telomerase and sirtuin 1 in all participants, male and female groups. There is evidence that sirtuin 1 delays telomere shortening by improving telomerase activity (Palacios et al. 2010). In women, telomerase was significantly correlated with DNA/RNA damage and AGEs, the levels of which did not change with age like telomerase. As expected, telomerase levels did not correlate with age.

In the literature, AGEs are considered an important marker of aging. AGEs are proinflammatory products of non-enzymatic glycation of nucleic acids, proteins, and lipids. They are endogenously formed during physiological and especially pathological conditions and can also be absorbed from the gastrointestinal tract as dietary AGEs (Rungratanawanich et al. 2021). AGEs are associated with numerous chronic diseases, diabetes, cardiovascular, neurological, and inflammatory diseases, the incidence and prevalence of which increase with age (Bodiga et al. 2013; Gaudioso et al. 2020).

In our study, the levels of AGEs depended on age. Significant differences were detected between under 30 and 35–50 groups. This result suggests that there is an age-dependent increase in the level of AGEs, but only up to a certain age, after which the level changes insignificantly. Our results are consistent with the results of other studies. It is known that the accumulation of AGEs is age dependent and associated with mitochondrial stress (Akhter et al. 2021). It can serve as a marker of cardiovascular diseases and its high levels are a risk factor for all-cause and especially cardiovascular mortality (Kilhovd et al. 2005; Sharifi-Zahabi et al. 2021).

Interestingly, there were differences in AGEs levels between the sexes. AGEs levels were significantly higher in men compared to females. The main difference was in the subgroup of 35–50. The study by Ebert et al. with 967 men and 812 women yielded similar results to our study. Plasma levels of AGEs were higher in males compared to females (Ebert et al. 2019). Tan et al. showed that males had higher serum levels of AGEs compared to females, which was also associated with increased levels of malondialdehyde and insulin resistance (Tan et al. 2011). We also found a positive correlation of AGEs and testosterone, which may indicate that men are more likely to have elevated AGEs levels because of higher levels of testosterone compared to women. Other correlations included a negative correlation between AGEs and GDF11 and a positive correlation with sirtuin 1. As already mentioned, the production of AGEs increases with age, whereas studies show that GDF11 levels decrease (discussed below). It is also well known that overproduction of AGEs is associated with DNA damage that can induce the expression of sirtuin 1, which is involved in DNA repair processes. Thus, higher AGEs may be the reason for sirtuin 1 production increase (Añón-Hidalgo et al. 2019; Alves-Fernandes and Jasiulionis 2019).

AGEs, along with other stimuli, induce inflammation and oxidative stress, processes associated with aging. Newly formed reactive oxygen species interact with macromolecules and modify their morphology and function. They also bind to nucleotides and alter DNA stability, affecting DNA transcription and repair, increasing the risk of *de novo* mutations. We evaluated the levels of DNA/RNA damage and showed that the DNA/RNA damage did not differ between groups (Borska et al. 2017).

DNA/RNA damage was negatively correlated with GDF11 in all groups and positively with sirtuin 1. This is the same pattern as in the case of AGEs. GDF11 decreases during aging and sirtuin 1 responds to DNA damage and is involved in DNA repair processes; therefore, DNA damage can induce the expression of sirtuin 1 as a compensatory mechanism (Alves-Fernandes and Jasiulionis 2019; Lagunas-Rangel 2019). It was negatively correlated with progesterone and positively correlated with DHEA, consistent with studies showing that oxidative stress and associated DNA damage is affected by sex hormones (Schiewer and Knudsen 2016).

Molecules belonging to the family of transforming growth factors are very interesting markers of aging. We analyzed the values of GDF15 and GDF11.

GDF15 is a molecule associated with stress and mitochondrial dysfunction. In our study, GDF15 was strongly age-dependent and independent of sex. Unsurprisingly, the lowest levels were in the under 35 group, but, unexpectedly, the highest levels were in the 35–50 group; then the levels slightly dropped but did not reach the low levels seen in the under 35 group. These changes in concentrations are not encountered in other studies. A gradual increase as a function of age is evident. In the study by Conte et al. , GDF15 levels increased age-dependently and negatively correlated with active lifestyle, even in the older person (Conte et al. 2020). Alcazar et al. also showed that age enhanced the production of GDF15, especially in older men. They also confirmed the association between GFD15 and sarcopenia and suggested that CDF15 can serve as a marker of frailty in older persons (Alcazar et al. 2021). Its levels are associated with increased mortality (Brenière et al. 2019; Freeman et al. 2020). In our study, we observed an atypical increase in middle-aged participants. GDF15 is also known to be a myokine and cardiokine, and its expression can be enhanced by extensive physical activity (Johann et al. 2021). It cannot be assumed that persons 35–50 years of age have higher physical activity than persons under 35 years of age. Therefore, there must be another mechanism that increased GDF15 levels in the 35–50 group. GDF15 correlated with age in all participants in the male group with BMI. Age and BMI are associated with pathological processes, including sarcopenia, which may increase GDF15 (Colleluori and Villareal 2021). Interestingly, the correlations depend on sex hormones. In the male and female groups, there was a negative correlation between progesterone and DHEA and GDF15; moreover, in the female group, we detected a negative correlation between estrogen and testosterone. Therefore, the decrease in sex hormones can be accompanied with the elevation of GDF15.

GDF11 is a regenerative rejuvenating factor. Its levels were highest in the group over 50. There was no difference between males and females, except in the 35–50 subgroup, where males had lower levels compared to females. Our results are not consistent with other studies. Añón-Hidalgo et al. confirmed that the levels of GDF11 age-dependently decreased (compared groups 41–50 and older), while they did not document any association between the levels of GFD11 and obesity or diabetes (Añón-Hidalgo et al. 2019). Similar results were published by Poggioli et al. They analyzed the levels of GDF11 in mammals (mice, rats, horses, sheep) (Poggioli et al. 2016). However, Schafer et al. described that the decline in GDF11 levels is associated with age-related diseases but not with healthy aging (Schafer et al. 2016). Moreover, Tanaka et al. proven that decreased levels of GDF11 in patients with COPD can be normalized by physical activity (Tanaka et al. 2021). Overall, the decline of GDF11 with chronological age and there its use as a potential biomarker of aging is questionable. Even in the scientific literature, new studies emerge with contradicting results. Possible explanations include different levels in healthy controls versus patients with metabolic diseases (depending on age) or non-specific measuring techniques as pointed out by Egerman (2019).

We revealed a negative correlation between GDF11 in the female group and sirtuin 1 in all groups. Jia et al. showed that GDF11 decreases steroidogenesis in human granulosa-lutein cells, which would be one reason for the negative correlation we found with progesterone (Jia et al. 2022). Regarding the correlation of GDF11 and sirtuin, our results are not consistent with other studies. Zhu et al. showed that GDF11 upregulated sirtuin 1 expression and activity, thus there should be a positive correlation (Zhu et al. 2021). Sirtuin 1 is a NAD^+^ dependent deacetylase that activity increases in the response to inflammation, metabolic stimuli, oxidative stress and DNA damage. Sirtuin 1 inhibits inflammation and plays a key role in mitochondrial biogenesis, thereby contributing to extending healthy life expectancy and reducing age-related diseases (Chuang et al. 2019).

In our study, sirtuin 1 levels declined with increasing age. The sirtuin 1 values were the lowest in the group over 50. Interestingly, the results of the studies are conflicting. Various chronic diseases are associated with decreased expression of sirtuin 1 (lung cancer, systemic sclerosis), while others are associated with an increase in sirtuin 1 levels (chronic kidney disease, Alzheimer’s disease) (Cornelius et al. 2013; Hosseninia et al. 2021; Zbroch et al. 2020; Manetti et al. 2022). Kilic et al. measured sirtuin 1 in children, adults and older people. Sirtuin 1 levels increased with age and positively correlated with age (Kilic et al. 2015). In contrast, Xu et al. confirmed that sirtuin 1 is reduced in senescent cells, including CD8 ^+^ CD28^−^ T cells from old donors, in which it is translocated to phagolysosomes and degraded (Xu et al. 2020). Stamatovic et al. revealed that sirtuin 1 expression is reduced in samples of aged mouse and human brains, which is associated with microvascular damage and disruption of the blood brain barrier (Stamatovic et al. 2019). Khanh et al. measured sirtuin 1 in mesenchymal stem cells from adipose tissue from infants and elderly. Expression was altered in the elderly person and induced senescence in the analyzed cells (Khanh et al. 2018). A similar pattern of expression described Kaur et al. who analyzed the expression of sirtuin 1 in endothelial progenitor cells derived from young and old individuals (below and over 60). The expression was higher in younger subjects compared to older subjects. Suppression of sirtuin 1 in cells from younger donors induced cell differentiation and senescence (Kaur et al. 2018). So there are studies that have reported results consistent with ours. Although we documented a decrease in sirtuin 1 values with age, we observed a negative correlation with age, especially in women. Only in the female group, sirtuin 1 correlated with estrogen, progesterone, testosterone, and DHEA. The expression of sirtuin 1 in human aortic endothelial cells is regulated by sex hormones. Thus, sirtuin 1 levels seem to be more affected in women, most likely by sex hormones and their fluctuations. Tsuchyia et al. demonstrated that DHEA, androstenedione, testosterone, estrone, and estradiol induce the expression of sirtuin 1 (Tsuchiya et al. 2020).

Another interesting result of our study was that NAD^+^ levels did not decrease with age and its level did not differ between groups. As expected, NAD^+^ is negatively correlated with above-mentioned sirtuin 1, NAD^+^ drives sirtuin 1 activity and is consumed by sirtuin 1. (Imai and Guarente 2016) There is a variety of studies that have shown a decline in NAD^+^ levels as a function of age and sex. Yang et al. analyzed blood samples from 1518 participants. The decrease in NAD^+^ levels was associated with aging but only in men, especially middle-aged (Yang et al. 2022). Massudi et al. and Clement et al. showed that the level of NAD^+^ decreased and negatively correlated with age in males as well as females (Massudi et al. 2012; Clement et al. 2019). Karas et al. showed that in healthy people the level of NAD^+^ negatively correlated with age (Karas et al. 2022). On the contrary, Schwartzmann et al. found that NAD^+^ did not depend on age, which corresponds to our results, but is influenced by sex. Women had higher levels than men (Schwarzmann et al. 2021). In our study, the level of NAD^+^ in women was slightly higher than in men as well.

Aging is associated with inflammaging, a process in which immune system imbalances occur. Changes in the number and function of immune cells cause low-grade damaging inflammation. The presence of inflammation can be monitored using many markers. One of these is the NLRP3 inflammasome. The assembly of the NLRP3 inflammasome is associated with the production of active IL-1beta and IL-18, prominent proinflammatory cytokines that play a role in inflammation. Aging is accompanied with NLRP3 activation (Gritsenko et al. 2020). Ablation of NLRP3 in mice can prevent age-related ovarian aging and thymic involution, while its presence drives thymic involution and immunosenescence (Navarro-Pando et al. 2021; Youm et al. 2012).

In our study, the levels of NLRP3 slightly increased and statistically differed between the group under 35 and over 50. Which is consistent with the results of other studies. Furthermore, we found a correlation between NLRP3 and age in the group of all participants and in the female group. In the female group, NLRP3 correlated with BMI. It is known that higher BMI is associated with inflammation (Cohen et al. 2021). Milan-Mattos et al. compared the production of proinflammatory cytokines in men and women. Older women had higher levels of cytokines compared to similarly aged men. (Milan-Mattos et al. 2019) In the male group, the correlations between NLRP3 and GDF11, GDF15, estrogen, and sirtuin 1 and a negative correlation with DNA/RNA damage were revealed. Using NLRP3 as a biomarker of aging is limited due to the fact that most of the values measured were extremely low.

Klotho is involved in numerous pathways associated with aging and its levels decline during aging (Buchanan et al. 2020). The levels of klotho did not differ between groups and subgroups but correlated with age in the group of all participants and in the female group which is consistent with other studies. Sanz et al. showed that low levels of klotho are associated with frailty in the elderly, whereas Mostafidi et al. confirmed that younger trained athletes had higher klotho than healthy non-athletic volunteers (Sanz et al. 2021; Mostafidi et al. 2016). Amaro-Gahete et al. evaluated the levels of klotho in middle aged men and women with sedentary lifestyle. Subjects in better physical condition had higher klotho values (Amaro-Gahete et al. 2019). Thus, it is evident that klotho levels change with age and physical activity and fitness. In the male group, klotho correlated with GDF11 and telomerase. Ullah et al. performed in vitro study on stem cells. Klotho deficiency reduced the activity of telomerase (Ullah et al. 2019). Thus, the increase in klotho is tied to an increase in other anti-aging parameters that tend to decrease with age.

## Conclusions

Our results are complex and detailed. They show that the levels of some selected markers vary with age, while others are more gender dependent. This confirms that aging is associated with changes in the processes in which the measured markers are involved and also occur differently in men and women. The findings can be used in clinical practice. Knowledge of the levels of markers and their changes captures the process of biological aging, its progression, and the risk of age-related diseases and death from any cause. Understanding the biological age is also important for initiating therapy in elderly patients.

### Supplementary Information

Below is the link to the electronic supplementary material.

Supplementary file1 (DOCX 45 KB)

## Data Availability

The data supporting published results are available from the cor-responding author if requested.

## References

[CR1] Akhter F, Chen D, Akhter A, Yan SF, Du YSS (2021). Age-dependent accumulation of dicarbonyls and advanced glycation endproducts (AGEs) associates with mitochondrial stress. Free Radic Biol Med.

[CR2] Alcazar J, Frandsen U, Prokhorova T (2021). Changes in systemic GDF15 across the adult lifespan and their impact on maximal muscle power: the Copenhagen Sarcopenia study. J Cachexia Sarcopenia Muscle.

[CR3] Alves-Fernandes DK, Jasiulionis MG (2019). The role of SIRT1 on DNA damage response and epigenetic alterations in cancer. Int J Mol Sci..

[CR4] Amaro-Gahete FJ, De-la-O A, Jurado-Fasoli L, Gutiérrez Á, Ruiz JR, Castillo MJ (2019). Association of physical activity and fitness with S-Klotho plasma levels in middle-aged sedentary adults: the FIT-AGEING study. Maturitas.

[CR5] Añón-Hidalgo J, Catalán V, Rodríguez A (2019). Circulating GDF11 levels are decreased with age but are unchanged with obesity and type 2 diabetes. Aging.

[CR6] Bodiga VL, Eda SR, Bodiga S (2013). Advanced glycation end products: role in pathology of diabetic cardiomyopathy. Hear Fail Rev.

[CR7] Borska L, Kremlacek J, Andrys C (2017). Systemic inflammation, oxidative damage to nucleic acids, and metabolic syndrome in the pathogenesis of psoriasis. Int J Mol Sci..

[CR8] Brenière C, Méloux A, Pédard M (2019). Growth Differentiation Factor-15 (GDF-15) is associated with mortality in ischemic stroke patients treated with acute revascularization therapy. Front Neurol..

[CR9] Buchanan S, Combet E, Stenvinkel P, Shiels PG (2020). Klotho, Aging, and the failing kidney. Front Endocrinol.

[CR10] Chaudhuri J, Bains Y, Guha S (2018). The role of advanced glycation end products in aging and metabolic diseases: bridging association and causality. Cell Metab.

[CR11] Chen L, Luo G, Liu Y (2021). Growth differentiation factor 11 attenuates cardiac ischemia reperfusion injury via enhancing mitochondrial biogenesis and telomerase activity. Cell Death Dis..

[CR12] Chuang YC, Chen SD, Jou SB (2019). Sirtuin 1 regulates mitochondrial biogenesis and provides an endogenous neuroprotective mechanism against seizure-induced neuronal cell death in the hippocampus following status epilepticus. Int J Mol Sci..

[CR13] Clemens Z, Sivakumar S, Pius A (2021). The biphasic and age-dependent impact of klotho on hallmarks of aging and skeletal muscle function. Elife..

[CR14] Clement J, Wong M, Poljak A, Sachdev P, Braidy N (2019). The plasma NAD+ metabolome is dysregulated in “normal” aging. Rejuvenation Res.

[CR15] Cohen E, Margalit I, Shochat T, Goldberg E, Krause I (2021). Markers of chronic inflammation in overweight and obese individuals and the role of gender: a cross-sectional study of a large cohort. J Inflamm Res.

[CR16] Colleluori G, Villareal DT (2021). Aging, obesity, sarcopenia and the effect of diet and exercise intervention. Exp Gerontol..

[CR17] Colloca G, Di Capua B, Bellieni A (2020). Biological and functional biomarkers of aging: definition, characteristics, and how they can impact everyday cancer treatment. Curr Oncol Rep.

[CR18] Conte M, Martucci M, Mosconi G (2020). GDF15 plasma level is inversely associated with level of physical activity and correlates with markers of inflammation and muscle weakness. Front Immunol..

[CR19] Cornelius C, Trovato Salinaro A, Scuto M (2013). Cellular stress response, sirtuins and UCP proteins in Alzheimer disease: role of vitagenes. Immun Ageing..

[CR20] Ebert H, Lacruz ME, Kluttig A (2019). Advanced glycation end products and their ratio to soluble receptor are associated with limitations in physical functioning only in women: results from the CARLA cohort. BMC Geriatr.

[CR21] Egerman MA, Glass DJ (2019). The role of GDF11 in aging and skeletal muscle, cardiac and bone homeostasis. Crit Rev Biochem Mol Biol..

[CR22] Freeman DW, Noren Hooten N, Kim Y (2020). Association between GDF15, poverty and mortality in urban middle-aged African American and white adults. PLoS One..

[CR23] Fujita Y, Taniguchi Y, Shinkai S, Tanaka M, Ito M (2016). Secreted growth differentiation factor 15 as a potential biomarker for mitochondrial dysfunctions in aging and age-related disorders. Geriatr Gerontol Int.

[CR24] Gaudioso G, Collotta D, Chiazza F (2020). Advanced glycation end products (AGEs) in metabolic disease: linking diet, inflammation and microbiota. Proc Nutr Soc..

[CR25] González-Dominguez A, Montañez R, Castejón-Vega B (2021). Inhibition of the NLRP3 inflammasome improves lifespan in animal murine model of Hutchinson-Gilford Progeria. EMBO Mol Med..

[CR26] Gonzalez-Hunt CP, Wadhwa M, Sanders LH (2018). DNA damage by oxidative stress: measurement strategies for two genomes. Curr Opin Toxicol.

[CR27] Gritsenko A, Green JP, Brough D, Lopez-Castejon G (2020). Mechanisms of NLRP3 priming in inflammaging and age related diseases. Cytokine Growth Factor Rev.

[CR29] Hartmann A, Hartmann C, Secci R, Hermann A, Fuellen G, Walter M (2021). Ranking Biomarkers of aging by citation profiling and effort scoring. Front Genet..

[CR30] Hartmann A, Hartmann C, Secci R, Hermann A, Fuellen G, Walter M (2021). Ranking biomarkers of aging by citation profiling and effort scoring. Front Genet..

[CR31] Hertzog RG, Popescu DM, Călborean O (2021). Telomerase level: a useful tool to predict longevity. J Aging Sci.

[CR32] Hosseninia S, Ameli A, Aslani MR, Pourfarzi F, Ghobadi H (2021). Serum levels of sirtuin-1 in patients with lung cancer and its association with karnofsky performance status. Acta Biomed..

[CR33] Imai SI, Guarente L (2014). NAD+ and sirtuins in aging and disease. Trends Cell Biol..

[CR34] Imai SI, Guarente L (2016). It takes two to tango: NAD+ and sirtuins in aging/longevity control. NPJ Aging Mech Dis.

[CR35] Jia Q, Liu B, Dang X (2022). Growth differentiation factor-11 downregulates steroidogenic acute regulatory protein expression through ALK5-mediated SMAD3 signaling pathway in human granulosa-lutein cells. Reprod Biol Endocrinol.

[CR36] Johann K, Kleinert M, Klaus S (2021). The role of gdf15 as a myomitokine. Cells..

[CR37] Jylhävä J, Pedersen NL, Hägg S (2017). Biol Age Predictors Ebiomed.

[CR38] Kane AE, Sinclair DA (2018). Sirtuins and NAD+ in the development and treatment of metabolic and cardiovascular diseases. Circ Res.

[CR39] Karas A, Holmannova D, Borsky P (2022). Significantly altered serum levels of NAD, AGE, RAGE, CRP, and elastin as potential biomarkers of psoriasis and aging—a case-control study. Biomedicines.

[CR40] Kaur I, Rawal P, Rohilla S (2018). Endothelial progenitor cells from aged subjects display decreased expression of sirtuin 1, angiogenic functions, and increased senescence. Cell Biol Int.

[CR41] Khanh VC, Zulkifli AF, Tokunaga C, Yamashita T, Hiramatsu Y, Ohneda O (2018). Aging impairs beige adipocyte differentiation of mesenchymal stem cells via the reduced expression of Sirtuin 1. Biochem Biophys Res Commun.

[CR42] Kidir V, Aynali A, Altuntas A (2017). Actividad de la telomerasa en pacientes en etapas de 2–5D con enfermedad renal crónica. Nefrol.

[CR43] Kilhovd BK, Juutilainen A, Lehto S (2005). High serum levels of advanced glycation end products predict increased coronary heart disease mortality in nondiabetic women but not in nondiabetic men: a population-based 18-year follow-up study. Arterioscler Thromb Vasc Biol.

[CR44] Kilic U, Gok O, Erenberk U (2015). A remarkable age-related increase in sirt1 protein expression against oxidative stress in elderly: SIRT1 gene variants and longevity in human. PLoS One..

[CR45] Lagunas-Rangel FA (2019). Current role of mammalian sirtuins in DNA repair. DNA Repair.

[CR46] Liu J, He MH, Peng J (2016). Tethering telomerase to telomeres increases genome instability and promotes chronological aging in yeast. Aging.

[CR47] López-Otín C, Blasco MA, Partridge L, Serrano M, Kroemer G (2022). Hallmarks of aging: an expanding universe. Cell..

[CR48] Manetti M, Rosa I, Fioretto BS, Matucci-Cerinic M, Romano E (2022). Decreased serum levels of SIRT1 and SIRT3 correlate with severity of skin and lung fibrosis and peripheral microvasculopathy in systemic sclerosis. J Clin Med.

[CR49] Massudi H, Grant R, Braidy N, Guest J, Farnsworth B, Guillemin GJ (2012). Age-associated changes in oxidative stress and NAD+ metabolism in human tissue. PLoS One..

[CR50] McNally EJ, Luncsford PJ, Armanios M (2019). Long telomeres and cancer risk: the price of cellular immortality. J Clin Invest.

[CR51] Milan-Mattos JC, Anibal FF, Perseguini NM (2019). Effects of natural aging and gender on pro-inflammatory markers. Brazilian J Med Biol Res..

[CR52] Mostafidi E, Moeen A, Nasri H, Hagjo AG, Ardalan M (2018). Serum klotho levels in trained athletes. Nephro-Urology Mon..

[CR53] Navarro-Pando JM, Alcocer-Gómez E, Castejón-Vega B (2021). Inhibition of the NLRP3 inflammasome prevents ovarian aging. Sci Adv..

[CR54] Niedrist T, Pailer S, Jahrbacher R, Gruber H-J, Herrmann M, Renner W (2022). Intensity-dependent stimulation of leukocyte telomerase activity by endurance exercise—a pilot study. LaboratoriumsMedizin.

[CR55] Noureen N, Wu S, Lv Y (2021). Integrated analysis of telomerase enzymatic activity unravels an association with cancer stemness and proliferation. Nat Commun.

[CR56] Palacios JA, Herranz D, De Bonis ML, Velasco S, Serrano M, Blasco MA (2010). SIRT1 contributes to telomere maintenance and augments global homologous recombination. J Cell Biol.

[CR58] Poggioli T, Vujic A, Yang P (2016). Circulating growth differentiation factor 11/8 levels decline with age. Circ Res.

[CR59] Preston J, Biddell B (2021). The physiology of ageing and how these changes affect older people. Medicine.

[CR60] Razgonova MP, Zakharenko AM, Golokhvast KS (2020). Telomerase and telomeres in aging theory and chronographic aging theory (review). Mol Med Rep.

[CR61] Rungratanawanich W, Qu Y, Wang X, Essa MM, Song BJ (2021). Advanced glycation end products (AGEs) and other adducts in aging-related diseases and alcohol-mediated tissue injury. Exp Mol Med.

[CR63] Sanz B, Arrieta H, Rezola-Pardo C (2021). Low serum klotho concentration is associated with worse cognition, psychological components of frailty, dependence, and falls in nursing home residents. Sci Reports..

[CR64] Schafer MJ, Atkinson EJ, Vanderboom PM (2016). Quantification of GDF11 and myostatin in human aging and cardiovascular disease. Cell Metab.

[CR65] Schiewer MJ, Knudsen KE (2016). Linking DNA damage and hormone signaling pathways in cancer. Trends Endocrinol Metab.

[CR66] Schwarzmann L, Pliquett RU, Simm A, Bartling B (2021). Biosci Rep..

[CR67] Sharifi-Zahabi E, Sharafabad FH, Abdollahzad H, Malekahmadi M, Rad NB (2021). Circulating advanced glycation end products and their soluble receptors in relation to all-cause and cardiovascular mortality: a systematic review and meta-analysis of prospective observational studies. Adv Nutr.

[CR68] Song L, Wu F, Li C, Zhang S (2022). Dietary intake of GDF11 delays the onset of several biomarkers of aging in male mice through anti-oxidant system via Smad2/3 pathway. Biogerontology.

[CR69] Stamatovic SM, Martinez-Revollar G, Hu A, Choi J, Keep RF, Andjelkovic AV (2019). Decline in Sirtuin-1 expression and activity plays a critical role in blood-brain barrier permeability in aging. Neurobiol Dis.

[CR70] Tan KCB, Shiu SWM, Wong Y, Tam X (2011). Serum advanced glycation end products (AGEs) are associated with insulin resistance. Diabetes Metab Res Rev.

[CR71] Tanaka R, Koarai A, Yamada M (2021). Longitudinal relationship between growth differentiation factor 11 and physical activity in chronic obstructive pulmonary disease. Int J COPD.

[CR72] Tsuchiya T, Takei A, Tsujikado K, Inukai T (2020). Effects of androgens and estrogens on sirtuin 1 gene expression in human aortic endothelial cells. Saudi Med J.

[CR73] Ullah M, Sun Z, Hare JM (2019). Klotho deficiency accelerates stem cells aging by impairing telomerase activity. J Gerontol—Ser A Biol Sci Med Sci.

[CR74] Vaiserman A, Krasnienkov D (2021). Telomere length as a marker of biological age: state-of-the-art, open issues, and future perspectives. Front Genet.

[CR75] Wang DX, Zhu XD, Ma XR (2021). Loss of growth differentiation factor 11 shortens telomere length by downregulating telomerase activity. Front Physiol.

[CR76] Wischhusen J, Melero I, Fridman WH (2020). Growth/differentiation factor-15 (GDF-15): from biomarker to novel targetable immune checkpoint. Front Immunol.

[CR77] Xu C, Wang L, Fozouni P (2020). SIRT1 is downregulated by autophagy in senescence and ageing. Nat Cell Biol.

[CR78] Yang F, Deng X, Yu Y (2022). Association of human whole blood NAD+ Contents with aging. Front Endocrinol.

[CR79] Youm YH, Kanneganti TD, Vandanmagsar B (2012). The NLRP3 inflammasome promotes age-related thymic demise and immunosenescence. Cell Rep.

[CR80] Zbroch E, Bazyluk A, Malyszko J (2020). The serum concentration of anti-aging proteins, sirtuin1 and αKlotho in patients with end-stage kidney disease on maintenance hemodialysis. Clin Interv Aging.

[CR81] Zhang C, Lin Y, Zhang K (2021). GDF11 enhances therapeutic functions of mesenchymal stem cells for angiogenesis. Stem Cell Res Ther.

[CR82] Zhao L, Zhang S, Cui J (2019). TERT assists GDF11 to rejuvenate senescent VEGFR2+/CD133+ cells in elderly patients with myocardial infarction. Lab Investig.

[CR83] Zhu HZ, Zhang LY, Zhai ME (2021). GDF11 alleviates pathological myocardial remodeling in diabetic cardiomyopathy through SIRT1-dependent regulation of oxidative stress and apoptosis. Front Cell Dev Biol.

