# Supplementary material for: Evaluation of potential aging biomarkers in healthy individuals: telomerase, AGEs, GDF11/15, sirtuin 1, NAD+, NLRP3, DNA/RNA damage, and klotho
Source: Biogerontology. 2023 Jul 31;24(6):937–55. doi: 10.1007/s10522-023-10054-x (PMC10615959; doi:10.1007/s10522-023-10054-x)
Supplement: Supplementary file 1 — Supplementary file1 (DOCX 45 KB) [file 10522_2023_10054_MOESM1_ESM.docx]

## Supplementary

Table 1 BMI and hormonal levels data of groups divided according to sex and sex and age

|  | N | Median | Q1 | Q3 | p value | Median | Q1 | Q3 | p value |
| --- | --- | --- | --- | --- | --- | --- | --- | --- | --- |
| BMI | | | | | | Estrogen pmol/l | | | |
| Female (F) | 84 | 24.96 | 22.66 | 28.55 | p<0.01 | 50.4 | 171.55 | 387.75 | p<0.05 |
| Male (M) | 85 | 27.59 | 25.28 | 29.36 |  | 87.5 | 108.60 | 131.80 |  |
| F < 35 | 30 | 24.20 | 22.28 | 27.35 | p<0.05 | 83.5 | 289.75 | 510.3 | p<0.05 |
| M <35 | 27 | 27.08 | 24.84 | 28.43 |  | 85.0 | 101.30 | 123.8 |  |
| F 35–50 | 28 | 26.73 | 23.91 | 29.82 | NS | 152.85 | 278.65 | 517.12 | p<0.0001 |
| M 35–50 | 31 | 27.42 | 25.65 | 30.13 |  | 87.05 | 115.30 | 140.45 |  |
| F > 50 | 26 | 25.04 | 23.31 | 27.09 | NS | 29.7 | 48.65 | 89.45 | p<0.01 |
| M > 50 | 27 | 27.76 | 25.70 | 29.06 |  | 91.3 | 103.10 | 129.35 |  |
| Progesterone nmol/l | | | | | | Testosterone nmol/l | | | |
| F | 84 | 0.35 | 0.70 | 2.92 | p<0.01 | 0.50 | 0.77 | 1.14 | p<0.0001 |
| M | 85 | 0.35 | 0.52 | 0.67 |  | 11.52 | 13.99 | 17.34 |  |
| F < 35 | 30 | 0.6 | 0.86 | 9.10 | p<0.01 | 0.78 | 1.10 | 1.36 | p<0.0001 |
| M <35 | 27 | 0.4 | 0.59 | 0.71 |  | 13.42 | 15.74 | 19.83 |  |
| F 35–50 | 28 | 0.48 | 01.07 | 8.29 | p<0.01 | 0.60 | 0.78 | 1.29 | p<0.0001 |
| M 35–50 | 31 | 0.40 | 0.53 | 0.70 |  | 11.16 | 13.99 | 16.90 |  |
| F > 50 | 26 | 0.26 | 0.36 | 0.68 | NS | 0.34 | 0.47 | 0.70 | p<0.001 |
| M> 50 | 27 | 0.33 | 0.40 | 0.56 |  | 11.66 | 13.06 | 14.75 |  |
| Dehydroepiandrosterone µmol/l | | | | | |  |  |  |  |
| F | 84 | 2.4 | 3.8 | 5.73 | p<0.0001 |  |  |  |  |
| M | 85 | 4.4 | 6.1 | 7.80 |  |  |  |  |  |
| F < 35 | 30 | 3.23 | 4.65 | 6.7 | p<0.01 |  |  |  |  |
| M <35 | 27 | 5.45 | 7.60 | 9.1 |  |  |  |  |  |
| F 35–50 | 28 | 3.20 | 4.65 | 06.03 | p<0.001 |  |  |  |  |
| M 35–50 | 31 | 5.35 | 6.80 | 7.55 |  |  |  |  |  |
| F > 50 | 26 | 1.65 | 2.5 | 3.27 | p<0.001 |  |  |  |  |
| M> 50 | 27 | 2.80 | 4.6 | 5.55 |  |  |  |  |  |

Table 2: Correlations between parameters in male and female groups

|  | Spearman rho | p value | Spearman rho | p value |
| --- | --- | --- | --- | --- |
| MALES | | | FEMALES | |
| **NLRP3** | | | | |
| ESG | 0.303 | 0.005 | xx | xx |
| Sirtuin | -0.219 | 0.046 | xx | xx |
| GDF11 | 0.392 | 0.251e-03 | xx | xx |
| GDF15 | 0.224 | 0.040 | xx | xx |
| DNA/RNA | -0.465 | 7.20e-06 | xx | xx |
| Telomerase | -0.304 | 0.005 | xx | xx |
| Klotho | 0.243 | 0.025 | xx | xx |
| Age | xx | xx | 0.217 | 0.047 |
| BMI | xx | xx | 0.295 | 0.006 |
| **KLOTHO** | | | | |
| GDF11 | 0.319 | 0.003 | xx | xx |
| Telomerase | -0.271 | 0.012 | xx | xx |
| **TELOMERASE** | | | | |
| ESG | -0.234 | 0.031 | xx | xx |
| Sirtuin | 0.596 | 2.738e-09 | 0.699 | 2.959e-13 |
| GDF11 | -0.440 | 3.131e-05 | -0.233 | 0.035 |
| DNA/RNA | 0.386 | 0.262e-03 | 0.454 | 1.444e-05 |
| AGE | 0.293 | 0.006 | 0.473 | 5.440e-06 |
| NAD | xx | xx | -0.296 | 0.006 |
| **AGE** | | | | |
| Sirtuin | 0.366 | 0.678e-03 | 0.387 | 0.334e-03 |
| GDF11 | -0.243 | 0.027 | -0.274 | 0.013 |
| **DNA/RNA** | | | | |
| BMI | 0.310 | 0.004 | xx | xx |
| Sirtuin | 0.233 | 0.034 | 0.395 | 0.243e-03 |
| GDF11 | -0.335 | 0.002 | xx | xx |
| **GDF15** | | | | |
| Age | 0.649 | 1.917e-11 | 0.486 | 2.708e-06 |
| BMI | 0.219 | 0.044 | xx | xx |
| PRGS | -0.249 | 0.022 | -0.323 | 0.003 |
| ESG | xx | xx | -0.415 | 8.811e-05 |
| TSTR | xx | xx | -0.355 | 0.937e-03 |
| DHEA | -0.334 | 0.002 | -0.414 | 9.077e-05 |
| Sirtuin | xx | xx | -0.231 | 0.036 |
| **GDF11** | | | | |
| Sirtuin | -0.513 | 7.238e-07 | -0.379 | 0.459e-03 |
| PRGS | xx | xx | -0.351 | 0.001 |
| NAD | xx | xx | 0.409 | 0.135e-03 |
| **NAD** | | | | |
| PRGS | xx | xx | -0.240 | 0.028 |
| Sirtuin | xx | xx | -0.316 | 0.004 |
| **SIRTUIN** | | | | |
| age | xx | xx | -0.317 | 0.004 |
| ESG | xx | xx | 0.225 | 0.042 |
| PRGS | xx | xx | 0.255 | 0.021 |
| TSTR | xx | xx | 0.309 | 0.005 |
| DHEA | xx | xx | 0.310 | 0.005 |

Legend: ESG, estrogen; PRGS, progesterone; TSTR, testosterone; DHEA, dehydroepiandrosterone

Fig. 1 Correlation among markers in the male and female group

| M | **N3** | **KLO** | **TEL** | **AGE** | **DNA** | **15** | **11** | **NAD** | **sir1** | F | **N3** | **KL** | **TEL** | **AGE** | **DNA** | **15** | **11** | **NAD** | **sir1** |
| --- | --- | --- | --- | --- | --- | --- | --- | --- | --- | --- | --- | --- | --- | --- | --- | --- | --- | --- | --- |
| age |  |  |  |  |  | *** |  |  |  |  | * | * |  |  |  | *** |  |  | ** |
| BMI |  |  |  |  | ** | * |  |  |  |  | ** |  |  |  |  |  |  |  |  |
| ESG | ** |  | * |  |  |  | * |  |  |  |  |  |  |  |  | *** |  |  | * |
| PRGS |  |  |  |  |  | * |  |  |  |  |  |  |  |  |  | ** | ** | * | * |
| TSTR |  |  |  |  |  |  |  |  |  |  |  |  |  |  |  | *** |  |  | ** |
| DHEA |  |  |  |  |  | ** |  |  |  |  |  |  |  |  |  | *** |  |  | ** |
| sirt1 | * |  | *** | *** | * |  | *** |  | x |  |  |  | *** | *** | *** | * | *** | ** | x |
| NAD |  |  |  |  |  |  |  | x |  |  |  |  | ** |  |  |  | *** | x |  |
| 11 | *** | ** | *** | * | ** |  | x |  |  |  |  |  | * | * |  |  | x |  |  |
| 15 | * |  |  |  |  | x |  |  |  |  |  |  |  |  |  | x |  |  |  |
| DNA | *** |  | *** |  | x |  |  |  |  |  |  |  | *** |  | x |  |  |  |  |
| AGE |  |  | ** | x |  |  |  |  |  |  |  |  | *** | x |  |  |  |  |  |
| TEL | ** | * | x |  |  |  |  |  |  |  |  |  | x |  |  |  |  |  |  |
| KLO | * | x |  |  |  |  |  |  |  |  |  | x |  |  |  |  |  |  |  |

Legend: *p < 0.05, ** p < 0.01, *** p < 0.001; red stars, negative correlation; M, males; F, females; xx, no correlation; N3, NLRP3; KLO, klotho; TEL, telomerase; DNA, DNA/RNA damage, 15, 11, GDF15/11; sirt1, sirtuin 1; ESG, estrogen; PRGS, progesterone; TSTR, testosterone; DHEA, dehydroepiandrosterone
